# Prevalence of anti-retinal autoantibodies in different stages of Age-related macular degeneration

**DOI:** 10.1186/1471-2415-14-154

**Published:** 2014-12-08

**Authors:** Grazyna Adamus, Emily Y Chew, Frederick L Ferris, Michael L Klein

**Affiliations:** Casey Eye Institute, Ocular Immunology Laboratory, L467AD, Oregon Health and Science University, 3181 SW Sam Jackson Pk Rd, Portland, OR 97239 USA; National Eye Institute, National Institutes of Health, Bethesda, MD 20892 USA

**Keywords:** Age-related macular degeneration, AREDS, Autoantibodies, Enolase, Antibody signature, Biomarker, Retina, Macula, Smoking, Arthritis

## Abstract

**Background:**

Age-related macular degeneration (AMD) is the leading cause of central vision loss in older adults. Anti-retinal autoantibodies (AAbs) have been found in individuals with AMD. The goal of the study was to determine the AAb specificity in different stages of AMD, and determine whether there is a prevalent AAb signature.

**Methods:**

Sera of 134 participants in the Age-related Eye Disease Study were analyzed for anti-retinal AAbs by western blotting. The subjects were classified by diagnostic subgroups based upon their clinical classification: No AMD, Intermediate AMD, and Late AMD - geographic atrophy (GA) and Late AMD - neovascular (NV).

**Results:**

The presence of anti-retinal AAb was detected in 58% patients with Intermediate and Late AMD, and 54% of those with no AMD. AAbs bound to fifteen different retinal antigens. Most individuals had 1 specific AAbs (67%), with the remainder having 2 to 4 different AAbs. Over 40% of patients with Intermediate AMD, and 46% of those with GA had anti-enolase AAbs, compared with 29% of individuals with NV and 29% with no AMD. Different AAbs signatures related to NV as compared to GA and/or Intermediate AMD were distinguished**.** Anti-40-kDa (10%) and 42-kDa (16%) autoantibodies were associated with Intermediate AMD, while anti-30-kDa AAbs (23%) were primarily present in GA. Anti-32-kDa (12%), 35-kDa (21%), and 60-kDa (8%) AAbs were more frequent in NV AMD.

**Conclusions:**

A unique AAb pattern for each of the disease subgroups was present when AMD progressed from the intermediate to the late forms of severity. Differences in the frequency of specific AAbs between AMD subgroups suggested that they may participate in pathogenicity of AMD. Further studies are necessary to confirm these observations in the larger cohort and individual AMD patients over time.

## Background

Age-related macular degeneration (AMD) is the leading cause of central vision loss in older adults [1, 2]. The etiology of AMD appears to be diverse, including age, genetic predisposition, diet, smoking, and other environmental risk factors [3, 4]. Considerable evidence supports a strong role for local inflammation including the accumulation of macrophages, lymphocytes, and mast cells found in association with both forms of late AMD: geographic atrophy (GA) and neovascular AMD [5–9]. Large drusen deposits in the macula (intermediate AMD) generally precede development of the late stages of AMD. The composition of these immunologically active drusen deposits includes proteins, lipids, complement, and other substances that may act as triggers for immune responses in the eye [7, 10]. In addition, the activation of complement factors and secondary mediators of inflammation such as cytokines and chemokines has been demonstrated in serum from AMD patients [3, 11–13]. These findings suggest the possible involvement of IL-22 and IL-17 in the inflammation that contributes to pathogenicity of AMD, and C5a may be one of the factors contributing to the elevated serum levels in AMD patients [14, 15].

There is increasing evidence for the presence of anti-retinal autoantibodies (AAbs) in association with AMD, although it is unclear whether such AAbs play an active role in the etiology of disease or if they are generated in a response to retinal injury from the underlying disease processes [16–21]. It is possible that both the generation of AAbs as well as the activation of complement could be responses to retinal damage/degradation [21–23]. Nevertheless, recent studies have demonstrated the presence of various AAbs in individuals with AMD, including anti-aldolase C, anti-pyruvate kinase isoform M2, anti-retinaldehyde binding protein 1, and anti-retinol binding protein 3 [16, 17, 20, 24]. The detection of AAbs could conceivably be important in subtyping the disease with specific antibody signatures (multiple antibody arrays), eventually helping define pathogenesis and optimum therapy. The goal of our study was to determine the AAb specificity in different stages of AMD, and prevalence of AAb signatures in relation to AMD severity.

## Methods

### Patients

Sera of 134 participants in the Age-related Eye Disease Study (AREDS) were selected for analysis. Detailed demographic and clinical information was obtained before blood samples were collected. Informed consent to participate in the Age-related Eye Disease Study was obtained from all participants. The study was approved by the Oregon Health and Science University Institutional Review Board (IRB#2421) and was conformed to the provisions of the Declaration of Helsinki.

**Figure 1:**
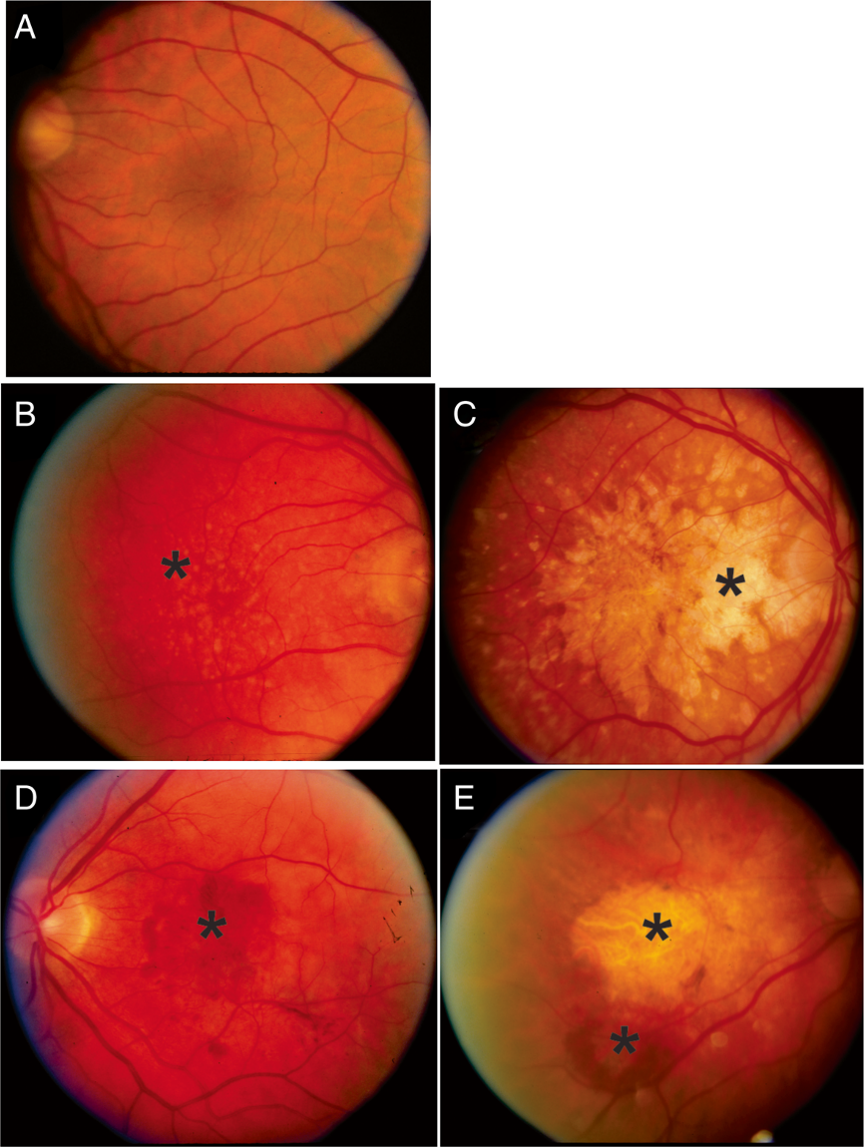
**Fundus photographs illustrating subgroups of AREDS patients in the study. (A)** No AMD; **(B)**. Intermediate AMD showing large drusen in the macula; **(C)** Late AMD - geographic atrophy; **(D)** Late AMD - neovascular AMD; **(E)** late AMD – both geographic atrophy and neovascular AMD. Stars indicate the affected areas.

All samples were stored at −80°C prior to use. Demographic characteristics of the study population are shown in Table 1. The study group consisted of 66 males and 68 females. The serum samples represented different disease stages reflecting AMD severity. The subjects were classified by diagnostic subgroups based upon their AREDS classification at the time of blood collection: 1. No AMD controls (N = 26) - no drusen or small drusen (<63 μm diameter) in the worse eye; 2. Intermediate AMD (N = 41) - large drusen (125 μm or greater) in one or both eyes; and 3. Late AMD (N = 67) – either geographic atrophy (N = 28), neovascular AMD (n = 33); or both neovascular AMD and geographic atrophy (N = 6). These AMD categories are illustrated in fundus photographs presented in Figure 1.

**Table 1:** **Association of autoantibodies against retinal proteins with different stages of AMD**

Stage of AMD		Number of subjects	Average age	Female/Male	Presence of AAbs seropositive/group
**No AMD (Control group)** None or small drusen		26	68	15/11	14/26 (54%)
**Intermediate AMD** Large drusen in one or both eyes (drusen 125 μm or greater)		41	69	25/16	26/41 (63%)
**Late AMD** Total (NV, GA, and Both)		67	73	33/34	38/67 (57%)
	**Geographic atrophy**	28	74	9/19	18/28 (64%)
	**Neovascular AMD**	33	72	19/14	16/33 (49%)
	**Both: neovascular and geographic atrophy**	6	73	5/1	4/6 (67%)

### Testing for anti-retinal autoantibodies

Western blotting was performed using retinal proteins extracted from a donor human retina with 1% octyl glucoside in phosphate/saline buffer (PBS), pH 7.2, and separated by gel electrophoresis as described previously [25]. Briefly, after separation by SDS-gel electrophoresis on a 10% gel (Bio-Rad) the proteins were transferred to an Immobilon membrane (Millipore). Next, individual strips containing retinal proteins were blocked with a buffer containing 10% normal goat serum and 1% bovine serum albumin in PBS for one hour followed by the incubation with 1:100 diluted serum for 1 hour. Secondary anti-human IgG (H and L chain) antibodies conjugated to alkaline phosphatase (Invitrogen) were added for another hour. Then color reaction was developed by adding the phosphatase substrate until dark bands appeared in positive controls (anti-recoverin and anti-enolase antibodies). A negative control strip was not incubated with primary antibodies.

### Statistical analysis

GraphPad Prism software (San Diego, CA) was used for statistical analysis. Statistical analyses were performed using one-way analysis of variance or Student’s *t* test. P <0.05 was considered as statistically significant. Fisher’s exact test was employed to evaluate differences in autoantibody frequency between groups. Differences between groups were evaluated using one-way ANOVA.

## Results

### Prevalence of autoantibodies in AMD subgroups

**Figure 2:**
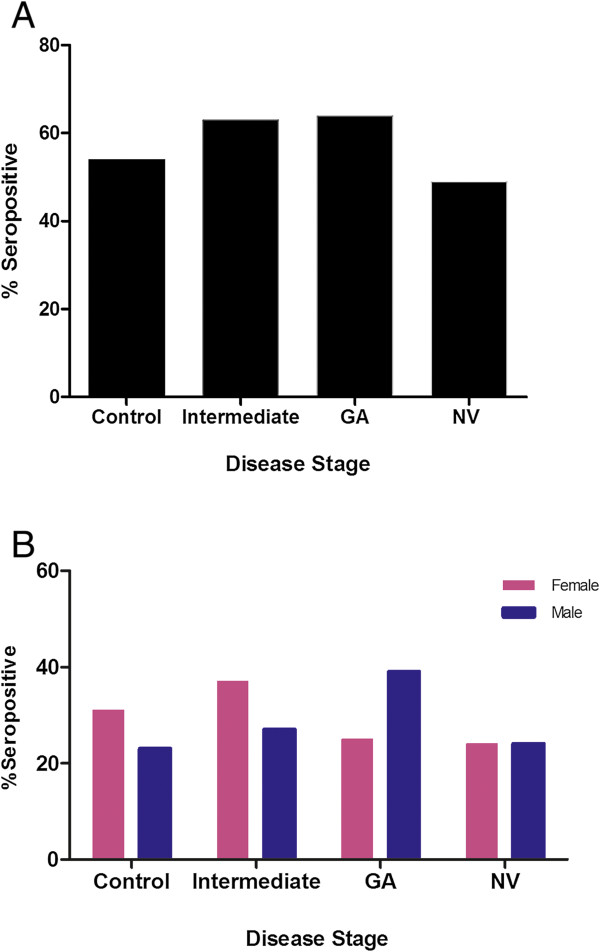
**Incidence of anti-retinal autoantibodies in patients with AMD and age-matched unaffected control subjects. (A)** Seropositive total subjects with AMD divided into the AMD subgroups and controls, and in **(B)** Seropositive females and males with AMD and controls. Groups: control group (no AMD), intermediate AMD, late AMD – geographic atrophy (GA), late AMD – neovascular (NV) AMD.

Sera of the 134 AREDS participants were analyzed for anti-retinal AAbs by western blotting using human retinal proteins. Table 1 shows the demographic characteristics and anti-retinal seropositivity for each AMD subgroup. Overall, the levels of AAbs were fairly constant (49% - 67%) over the severity spectrum of the disease (Table 1, Figure 2A). However, there were specific AAbs associated with different severity stages (see below). AAbs in females and males showed a similar tendency of almost equal occurrence with late AMD and showed only minor difference in incidence within severity disease stages (Figure 2B). The relatively persistent rate of anti-retinal AAbs across all subgroups suggests that autoantibodies were likely generated during the early stage of maculopathy or were a part of the aging process since the patients and controls were of similar age [26].

### Autoantibody signatures

**Figure 3:**
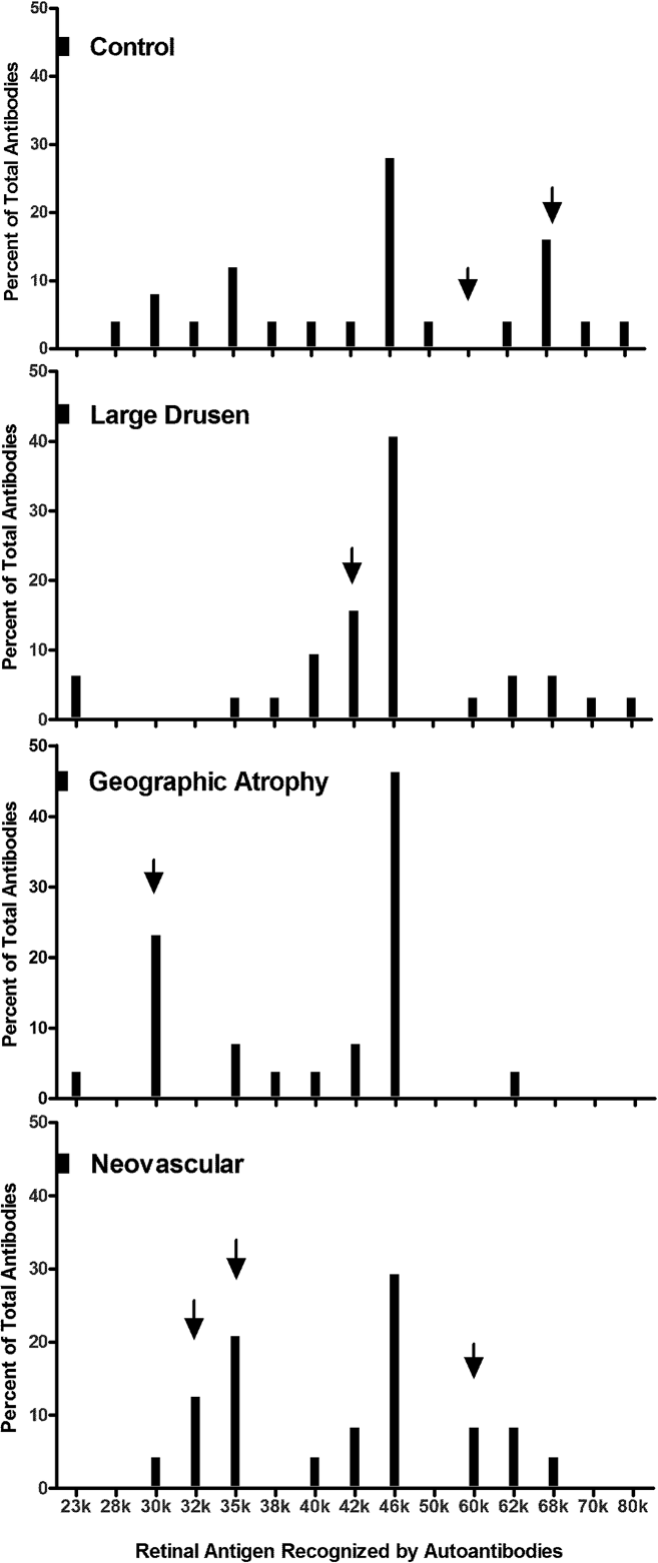
**Anti-retinal autoantibodies in different stages of AMD.** Distribution of anti-retinal autoantibodies against 15 different retinal proteins identified by molecular weight in the 4 study groups of patients: control group (no AMD), intermediate AMD, late AMD - geographic atrophy, and late AMD - neovascular AMD. Bars show the percent of positive AAbs for each antigen; arrows point at unique antigens for each AMD group.

AMD and age-control autoantibodies bound to 15 different retinal antigens were identified here by their molecular weight. Sixty nine percent of sera reacted with a singular antigen while the remaining 31% reacted with 2 to 4 different retinal antigens. In the AMD subgroups, 51% (21/41) of patients with intermediate AMD, 32% (9/28) of patients with late AMD-GA, and 30% (10/33) of patients with late AMD-NV were seropositive for 1 retinal antigen. In contrast, 19% (5/26) of control sera were positive for the singular AAb. Figure 3 shows the distribution of anti-retinal AAbs in each of the severity stages. Different AAb signatures related to intermediate AMD, as compared to late AMD (GA and/or NV) were distinguished, and there was a unique AAb pattern for each of the disease subgroups that changed when progressing from the intermediate to the late forms of AMD. The comparison of subgroups showed significant differences in the AAbs occurrence between AMD groups (p < 0.0001, one way ANOVA).

We evaluated the occurrence of specific antibodies for each AMD subgroup. We do not provide the identity of the targeted antigens (except enolase, which is a 46-kDa protein) because of insufficient serum amounts for full identification of these retinal antigens. Figure 3 illustrates AAb occurrence in each subgroup and Table 2 summarizes the major AAbs associated with different stages. We found that anti-40-kDa (10%) and 42-kDa (16%) autoantibodies were associated more commonly with intermediate AMD, while anti-30-kDa AAbs (23%) were primarily present in late AMD-GA. AAbs against 32-kDa (13%), 35-kDa (21%), and 60-kDa (8%) proteins were more frequent in individuals with late AMD-NV. Over 40% of patients with intermediate AMD, and 46% of those with late AMD-GA had ~1.5-fold higher rate of anti-enolase (anti-46-kDa) AAbs, compared with 29% of individuals with late AMD-NV and 28% of controls.

**Table 2:** **Seropositivity associated with different stages of AMD and control group**

AMD stage	Total seropositivity	Patients seropositive for a single antigen	Unique antigens*
**Controls**	14/26 (54%)	5/26 (19%)	35 k (12%)
			68 k (16%)
			46 k (28%)
**Intermediate AMD**	26/41 (63%)	21/41 (51%)	40 k (10%)
			**42 k (16%)**
			46 k (41%)
**Late AMD - Geographic Atrophy**	18/28 (64%)	9/28 (32%)	**30 k (23%)**
			46 k (46%)
**Late AMD - Neovascular AMD**	16/33 (49%)	10/33 (30%)	32 k (13%)
			**35 k (21%)**
			46 k (29%)
			60 k (8%)
**Late AMD - Both GA + NV**	4/6 (67%)	4/6 (67%)	Not determined

*Unique retinal antigens are presented as a percentage of total recognized antigens present within a group.Antigens are identified by molecular weight (K = 1000).

**Figure 4:**
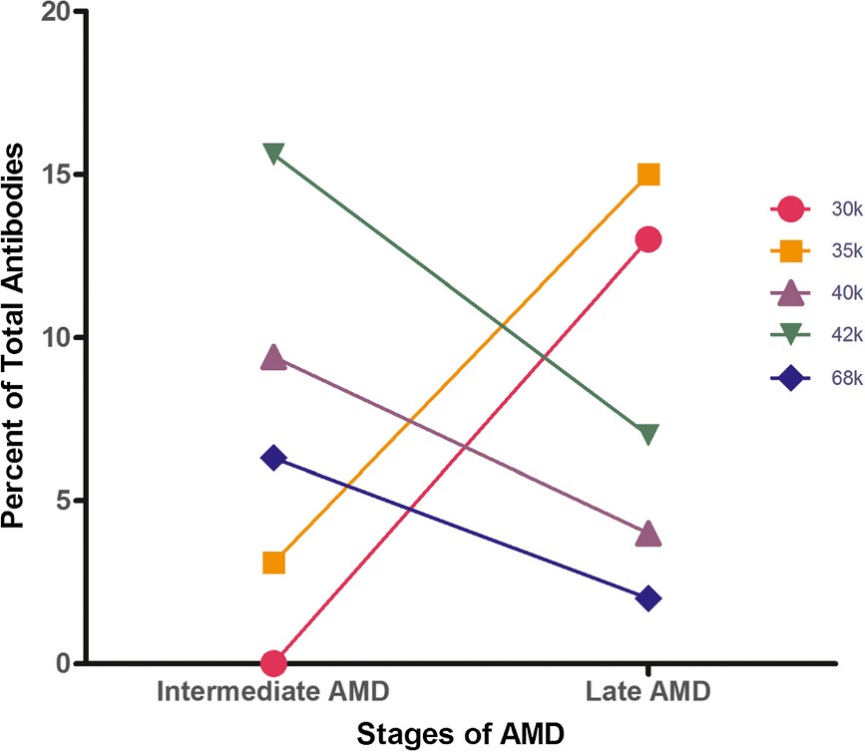
**Trends in AAbs associations between intermediate and late AMD.** Five anti-retinal AAbs designated by their target antigen molecular weight (30-kDa, 35-kDa, 40-kDa, 42-kDa, and 68-kDa) have a tendency to decrease in advance stages of AMD (anti-40-kDa, anti-42-kDa, and anti-68-kDa) as others to increase (anti-30-kDa and anti-35-kDa) that could potentially be used as disease biomarkers.

A similar tendency of decreasing serum AAbs in the late stages of AMD was observed for anti-42-kDa reactivity showing a 2-fold decline from 16% for intermediate AMD to 8% in late AMD-NV. Also, anti-42-kDa AAbs were 4-fold higher in the intermediate AMD group than in no AMD controls, in which only 4% of those AAbs were present. In contrast, anti-35-kDa AAbs showed ~7-fold increase in the late AMD-NV group as compared with the intermediate AMD group. The comparison of those 3 groups show statistical significance (one-way ANOVA, p = 0.0008). Figure 4 shows an increased level of anti-40-kDa, anti-42-Da, and anti-68-kDa AAbs in the intermediate AMD group, while anti-30-kDa and anti-35-kDa AAbs were elevated in late AMD. Together, these results suggest that some of those AAbs may potentially be considered as biomarkers for different stages of AMD.

### Association of autoantibodies with smoking and arthritis in AMD

**Figure 5:**
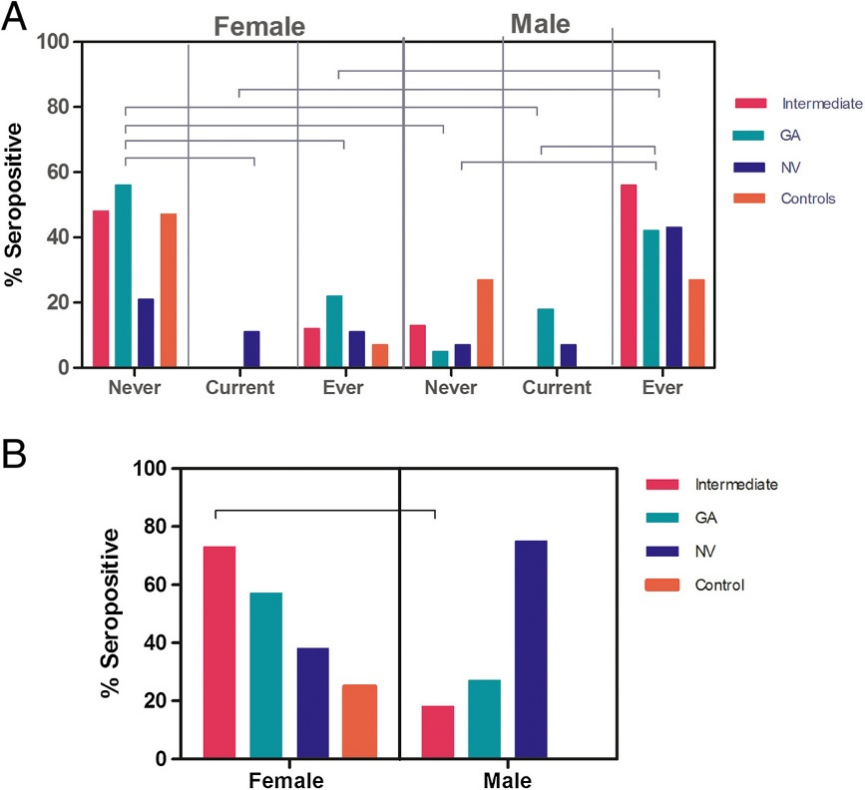
**Relationship of anti-retinal autoantibodies in different stages of AMD with smoking and arthritis. (A)** Prevalence of anti-retinal autoantibodies in men and women who never smoked, are current smokers, or who stopped smoking in AMD and control subjects. High anti-retinal seropositivity is observed in never smoking females that dropped 2 folds in ever smoker female patients. In contrast, smoking or discontinuation of smoking is associated with an increased frequency of AAbs in men regardless of the stage of disease. Never = never smoking female/male; Current = currently smoking female/male; Ever = former smoking female/male. **(B)** Differences in anti-retinal autoantibody association in female and male with AMD and arthritis and compared to the age-matched unaffected controls. Bars represent a percent of seropositive subjects in each subgroup. Note that female with intermediate AMD had 4 times more AAbs than men, in contrast men with late AMD-NV had 2 times higher frequency of anti-retinal antibodies. Horizontal lines show statistical significance between groups, p < 0.05 (One-way analysis of variance, Bonferroni’s multiple comparison test).

Genetic and environmental factors play a role as indicators of disease outcome in AMD, including smoking and arthritis [4, 27]. Smoking has been shown to predispose to the development of several AAbs [28, 29]. To determine whether current tobacco smokers, ex-smokers, and ever-smokers of both sexes with AMD have anti-retinal AAbs we analyzed antibody presence in three groups: 1) ever-smokers (n = 41), 2) current smokers (n = 8), or 3) former smokers (n = 59). Our results show that women ≤65 years that never smoked had significantly higher levels of AAbs than did men of the same age and a history of cigarette smoking. Gender differences remained in ex-smokers but with opposite trends in all stages of AMD. Figure 5A shows that never smoking women with intermediate AMD and AMD-GA had ~3 times higher prevalence of AAbs than never smoking men (Figure 5B). In contrast, current or ever male smokers had higher incidence of serum AAbs than women. Seropositivity in arthritis and AMD was found in 20/58 (35%) women and 11/50 (20%) men. Female with intermediate AMD had 4 times greater incidence of AAbs than men, and in contrast, men with NV had 2-fold greater frequency of anti-retinal autoantibodies than women. These differences were not statistically significant but the sample size was small.

## Discussion

The etiology of AMD is complex and includes genetic risk factors, environmental factors, age, and immune and autoimmune causes [30]. Several potential immune mediators are known to play a role in the pathogenic process of AMD, such as infiltration of macrophages, presence of cytokines/chemokines, T-lymphocytes, as well as formation of autoantibodies [1, 5]. Our studies demonstrated a complex pattern of AAbs against several retinal proteins in individuals with AMD with fairly constant presence over the severity spectrum of the disease. We also identified AAbs that could be markers of disease activity (Figures 3 and 4). We believe that early generation of anti-retinal AAbs may create an environment that promotes cell loss, angiogenesis, and progression to late stages of AMD [31]. Since in autoimmune diseases, the immune response is itself part of the disease process, it is possible that the consistent presence of AAbs suggests their contribution to pathogenic processes through the availability of the antigens from degenerating retina.

Previously published studies showed the association of AAbs with AMD, suggesting a contribution of the immune system to pathogenicity of the disease [7, 17, 19, 32, 33]. Several autoantibodies to retinal antigens have been identified, including anti-glial fibrillary acidic protein (GFAP), anti-α-enolase, and anti-carboxyethylpyrrole (CEP), an oxidized component of drusen [17, 32, 34, 35]. GFAP is a 52-kDa antigen, the main intermediate filament protein in mature astrocytes, that was targeted in 44% of the AMD patients’ population studied [17]; however, we have not found AAbs against 52-kDa antigen in a measurable level in our cohort. The study examined approximately the same number of patients as our study reported anti-enolase AAbs in 67% of the AMD patients, which are similar to our findings [17]. Other studies showed that AAbs in individuals with neovascular AMD recognized retinol-binding protein 3 (RBP3, 120-kDa), retinol-binding protein 1 (RLBP1, 36-kDa) and aldolase C (39-kDa) [20]. RBP3 is an essential protein for the exchange of retinoid between the RPE and photoreceptors and their survival because it prevents the potentially cytotoxic effects of retinoids. Autoantibodies against RBP3 were detected in 33% (6 of 18) of patients with AMD and also in 24% (11 of 45) of patients with Macular Telangiectasia Type 2 [36]. The authors suggested that the existence of mutual AAbs in MacTel-2 and AMD shared some common etiologic or pathogenic mechanisms for both conditions. RBP3, also known as interphotoreceptor retinoid binding protein (IRBP), is a unique protein to the photoreceptor cells in the retina, which has been found to be highly pathogenic in animals. Immunization of animals with IRBP induced an intraocular inflammatory disease that is primarily mediated by T cells but anti-IRBP antibodies are also present, suggesting their role in inflammation. Moreover, anti-IRBP autoantibodies and T cell have been also found in patients with uveitis, retinitis pigmentosa, and progressive rod-cone degeneration [37–41]. Cellular retinaldehyde-binding protein (CRALBP), transcribed from the RLBP1 gene, is a 36-kDa protein found in the RPE and in retinal Müller cells [42]. Both elevated seroreactivity to RBP3 and RLBP1 in AMD patients suggests that the inflammation, in particular, autoimmunity, is strongly associated with the pathogenesis of the disease [11]. In our cohort, a 35-kDa antigen is likely to be CRALBP although its identity wasn’t confirmed (due to insufficient quantity of samples) if confirmed, these AAbs would be found to be 2 times more frequent in individuals with AMD than in controls. Nonetheless, anti-CRALBP AAbs have been found in uveitis, showing 54% seropositivity in patients with uveitis compared to 17% in normal subjects [43], they were also detected in patients with cancer-associated retinopathy, CAR (Adamus, unpublished information). Altogether, these findings indicated that anti-IRBP and anti-CRALBP AAbs are not highly specific for AMD individuals.

It is not surprising to find autoantibodies against α-enolase (46-kDa), aldolase C (40-kDa), and pyruvate kinase M2 (60-62-kDa) that previously were found in both neovascular and geographic atrophy AMD [17, 20]. These key enzymes of the glycolytic pathway may promote autoimmunity by acting as autoantigens. α-Enolase, also called non-neuronal enolase, belongs to a family of glycolytic enzymes but also has other cellular functions related to its subcellular localizations that are distinct from its well-established activity in glycolysis [44–46]. Furthermore, differential expression of α-enolase and presence of specific AAbs have been related to several pathologies, such as cancer, Alzheimer’s disease, autoimmune diseases, and rheumatoid arthritis, among others. Antibodies against α-enolase have been strongly associated with CAR [25, 47–49]. Recently, we showed that AAbs against anti-glycolytic enzymes were highly associated with CAR and gynecological cancers [50]. In vitro and in vivo studies showed that anti-enolase AAbs have pathogenic potential in killing retinal cells [51–53]. Generation of AAbs against enolase and other anti-glycolytic enzymes may be a normal process since they can be found in healthy individuals. However, it is possible that excessive production of such autoantibodies can be generated as a consequence of enolase uptake by antigen-presenting cells and subsequent B cell activation, can potentially initiate a tissue injury as a result of immune complex deposition, or an induction of apoptosis leading to the death of retinal cells.

Autoantibodies against CEP, an adduct that develops from an oxidation fragment of docosahexaenoic acid (DHA), are present in plasma and are more abundant in AMD than in controls, suggesting their potential as a biomarker for AMD [32]. Animal studies showed that mice immunized with CEP-modified mouse serum albumin generated anti-CEP antibodies that consequently induced AMD-like lesions in the outer retina, suggesting that autoimmunity was associated with the initiation or progression of AMD [34]. CEP is typically present in photoreceptor rod outer segments, and RPE in the mouse retina, and its reactivity is more intense in photoreceptors of human AMD retina than healthy retina [32].

In AREDS, persons with either intermediate drusen, extensive small drusen, or the pigment abnormalities associated with AMD were more likely to be female, and more likely to have a history of arthritis [4]. This might suggest an association with chronic inflammatory disease. We found that women with arthritis and intermediate AMD had significantly greater incidence of AAbs than men, suggesting possible sex hormone effects. Changes in sex hormones mainly influence onset of rheumatoid arthritis in older individuals of both sexes [54]. Lower levels of testosterone in men, and early menopause in women, may be signs of premature aging, putting them at greater risk of developing diseases and thus antibodies.

Epidemiologic evidence indicates that smoking cigarettes results in an increased risk of AMD [27, 55]. Smokers of both sexes have an increased risk of developing seropositive rheumatoid arthritis (RA) [56]. For example, in RA, the presence of anti-citrullinated protein antibodies has been described to be specifically associated with smoking. Also, smoking has also been shown to be associated with AAbs in various other autoimmune diseases, such as anti-dsDNA in systemic lupus erythematosus, and anti-Jo1 in idiopathic inflammatory myopathy. There is evidence that citrullinated α-enolase is present in the lungs of smokers [57]. However, the precise mechanisms whereby smoking may trigger immunity to citrullinated α-enolase remain to be elucidated. Gender differences in our cohort were evident in AAb frequencies in never, current, and ever smokers with somewhat surprising results. Women who had never smoked presented with a higher prevalence of anti-retinal AAbs than women who were former smokers, which suggests a possible influence of sex hormones (e.g., estrogens). Estrogen strongly stimulate the immune response [58] and also can induce AAb levels in persons after quitting smoking [59]. Never-smoking men with AMD were protected from developing anti-retinal AAbs, in contrast to ex-smokers who had higher prevalence of AAbs. Testosterone plays an important role in the modulation of inflammatory processes and may neutralize the adverse changes of immune response [60]. Testosterone is known to promote apoptosis of Th2 cells and development of the Th1 phenotype of cytokine production, which results in suppression of humoral immune response. A fall of testosterone concentration in aging men can influence oxidative modification and the immune response, which is important in the pathogenesis of atherosclerosis.

## Conclusions

The role of AAbs in the induction or acceleration of retinal deterioration is uncertain. Our studies showed that AAbs against retinal proteins are apparently different in various AMD severity subgroups. However, the differences in frequency of specific AAbs between AMD subgroups may suggest that they participate in pathogenicity of AMD. Also, different AAbs co-exist in individual patients, possibly creating antibody signatures specific to each stage of AMD. If confirmed by other studies, these elevated levels of specific antibodies may be a useful predictor or biomarker of AMD progression from intermediate to late AMD. On the other hand, it is reasonable to suggest that the high frequency of AAbs in persons with AMD as well as healthy individuals is a consequence of this progressive ‘aging’ of the immune system. However, pathogenic AAbs that can be detected in peripheral blood years before the destruction of retinal cells may lead to obvious clinical symptoms. Regardless of their role, AAbs may be useful as biomarkers and we presented a few possible candidates for each stage of AMD progression even though we could not provide the identity of the targeted antigens (except enolase). This was a limitation of this research because insufficient serum amounts did not allow for full identification of these retinal antigens. Further studies are necessary to confirm our results in the larger cohort of patients with AMD, and also to examine individual patients over the progression of their macular disease, and to identify the targeted antigens.
